# Contribution of ferrous iron transporter FeoB to iron utilization, membrane stability and biofilm formation in *Aeromonas hydrophila*

**DOI:** 10.71150/jm.2605004

**Published:** 2026-10-31

**Authors:** Zimeng Zhang, Wei Su, Murtala Muhammad, Yuya Wang, Xinyan You, Fengjuan Ren, Muwei Li, Jiansong Ju, Baohua Zhao, Dong Liu

**Affiliations:** 1 College of Life Science, Hebei Normal University, Shijiazhuang 050024, China; 2 Department of Clinical Laboratory, Affiliated Hospital of Hebei University, Baoding 071000, China; 3 Department of Biochemistry, Kano University of Science and Technology, Wudil 713281, Nigeria

**Keywords:** FeoB, *Aeromonas hydrophila*, iron homeostasis, ferrous iron transporter, biofilm formation, DIA proteomics

## Abstract

*Aeromonas hydrophila* (*A. hydrophila*) is an infectious agent that can infect both fish and humans, posing significant threats to aquaculture and public health. Iron acquisition is essential for bacterial survival, colonization, and pathogenicity, as it is crucial to many metabolic and enzymatic processes. Ferrous iron transporter B (FeoB) is a major component of the bacterial iron uptake system, mediating the transport of ferrous iron (Fe^2+^) and playing a key role in cellular iron homeostasis. The present study elucidates the role of FeoB in iron utilization, bacterial physiology, and pathogenicity of *A. hydrophila* ATCC 7966. A stable *feoB*-deleted mutant strain (AhΔ*feoB*) and its complemented strain (CΔ*feoB*) were constructed. Our results demonstrated that deletion of *feoB* impaired ferrous‑iron accumulation and substrate affinity, significantly increased sensitivity to clindamycin, sodium deoxycholate, and oxidative stress, reduced biofilm formation, and downregulated biofilm-associated genes. Furthermore, loss of the *feoB* gene resulted in abnormal cellular morphology and altered expression of 12 out of 15 tested virulence-related genes. Unlike in previous reports, AhΔ*feoB* showed no significant differences in growth characteristics, hemolytic activity and virulence in a BALB/c mouse infection model under iron-limited conditions. Moreover, DIA-based proteomic analysis revealed that *feoB* is a core protein essential for iron homeostasis, and its deletion profoundly impacts bacterial metabolism, adaptability, and iron transport. These findings emphasize the central role of FeoB in iron homeostasis and in the regulation of critical physiological functions in *A. hydrophila* ATCC 7966.

## Introduction

Iron is an essential micronutrient for bacterial pathogens, acting as a critical cofactor for enzymes and proteins involved in electron transport, DNA replication, oxidative phosphorylation, and various metabolic processes (Guan et al., 2025; Shadangi et al., 2025). However, in the host microenvironment, free iron availability is extremely limited due to sequestration in ferric (Fe³⁺) form by a host defense strategy termed nutritional immunity (Hood and Skaar, 2012). Pathogenic bacteria have evolved diverse iron acquisition systems to overcome iron limitation, among which the ferrous iron (Fe^2+^) transport system encoded by the *feo* operon, which produces three core proteins (FeoA, FeoB, and FeoC). FeoB acts as an indispensable transmembrane transporter and the functional core of the system, and it is one of the most conserved and predominantly functions under anaerobic or microaerophilic conditions (Gómez-Garzón et al., 2022; Lau et al., 2016; Shin et al., 2020). FeoB plays a critical role in bacterial virulence, environmental adaptability, and stress resistance. In Gram-negative pathogens including *Escherichia coli*, *Salmonella enterica*, *Helicobacter pylori*, *Aeromonas veronii*, and *Riemerella anatipestifer*, *feoB* deletion impairs host colonization, intracellular survival, oxidative stress tolerance, biofilm formation and in vivo pathogenicity (Guan et al., 2025; Huang et al., 2023; Kammler et al., 1993; Naikare et al., 2006; Tsolis et al., 1996; Velayudhan et al., 2000).

*Aeromonas hydrophila* is a Gram-negative opportunistic pathogen that infects aquatic organisms and mammals, including humans, causing severe aquatic animal diseases and human clinical infections (Chen et al., 2026). Its high environmental adaptability and pathogenicity rely on efficient iron acquisition systems to survive in iron-limited environments and host niches (Lange et al., 2020). Moreover, regulation of iron homeostasis is a well-recognized determinant of its virulence (Teng et al., 2018). However, the biological role and regulatory mechanism of FeoB-mediated Fe^2+^ transport in *A. hydrophila* remain uncharacterized. Although FeoB has been verified to modulate motility, biofilm formation, adhesion, invasion capability and virulence in *Aeromonas veronii*, significant interspecific differences in iron acquisition systems, Fur regulatory patterns and virulence mechanisms exist between *A. veronii* and *A. hydrophila* (Guan et al., 2025; Ran et al., 2018). The biological function and molecular mechanism of FeoB-mediated Fe^2+^ transport in *A. hydrophila* remain unclear.

In this study, a stable *feoB* deletion mutant (AhΔ*feoB*) and its complemented strain were constructed in *A. hydrophila* ATCC 7966 via homologous recombination. Phenotypic assays were performed to investigate the roles of *feoB* in bacterial growth, iron utilization, motility, hemolytic activity, stress tolerance, antibiotic resistance, and biofilm formation. Additionally, BALB/c mouse infection models, quantitative real-time PCR (qRT-PCR) and data-independent acquisition (DIA) quantitative proteomics were applied to explore the regulatory mechanism of FeoB in bacterial virulence.

This study clarifies the essential functions of *feoB* in iron homeostasis and pathogenicity, providing a promising target for novel anti-*A. hydrophila* antimicrobial development.

## Materials and Methods

### Strains, plasmids, and culture conditions

*A. hydrophila* ATCC 7966, *Escherichia coli* DH5α and *E. coli* S17-λpir were used in this study. *A. hydrophila* was cultured at 30°C in Luria–Bertani (LB) medium, *E. coli* strains were maintained at 37°C. The plasmids pRE112 and pBBR1MCS1 were used for gene deletion and genetic complementation, respectively. Ampicillin (Amp, 50 μg/ml) was supplemented when necessary (Table 1).

### Experimental animals, anesthesia, and euthanasia

Female BALB/c mice (4–6 weeks old) were obtained from the Hebei CDC (Shijiazhuang, China) and maintained at 23°C, under a 12 h light/dark cycle with free access to food and water throughout the experiment. All animal procedures were approved by the Animal Ethics Committee of Hebei Normal University (IACUC No. LLSC2025091) and conducted in accordance with the guidelines of Hebei Province experimental animal care, China. Mice were euthanized via intraperitoneal injection of pentobarbital sodium (50 mg/kg) and euthanized by cervical dislocation to minimize suffering.

### Generation and verification of the *feoB* mutant and complemented strains

The *feoB*-deficient mutant (AhΔ*feoB*) was generated via homologous recombination using the suicide vector pRE112. The flanking regions of *feoB* gene (1,014 bp) were PCR-amplified from *A. hydrophila* genomic DNA, yielding a fragment containing 446 bp upstream of the translational start site (ATG) and 568 bp downstream of the stop codon (TCA). The amplified fragment was cloned into pRE112 to generate the recombinant construct pAR1. After verification by sequencing, pAR1 was introduced into *A. hydrophila* via conjugation (Li et al., 2023b). The feoB-deficient mutant was obtained by double crossover homologous recombination and confirmed by PCR (Zhao et al., 2022).

For complementation, the full-length *feoB* open reading frame was amplified and cloned into pBBR1MCS1. The recombinant plasmid pBR-*feoB* was then electroporated into AhΔ*feoB* to generate the complemented strain CΔ*feoB* (Wang et al., 2020).

### Growth curve assessment

The growth profiles of WT, AhΔ*feoB*, and CΔ*feoB* were evaluated in LB media using the Bioscreen C automated growth analyzer. Overnight cultures were normalized to equal cell densities with or without 0.6 mM 2,2'-dipyridyl (DIP) (Teng et al., 2018). For iron stress assays, strains were cultivated in M9 minimal medium with or without 0.2 mM FeSO_4_ supplementation, as described previously (Brown et al., 2023), while uninoculated LB medium served as the blank control. OD_600_ values were measured at 2 h intervals and bacterial cell counts were determined by colony-forming unit (CFU) enumeration (Muhammad et al., 2020).

### Motility and hemolysis evaluation

Swimming and swarming motilities were examined using 0.25% (w/v) LB agar and 0.35% (w/v) swarm agar, respectively. The motility was measured by examining the diameter of bacterial motility from the center toward the periphery of the plates (Liu et al., 2025b). The hemolytic activity was evaluated by inoculating onto sheep blood agar plates at 30℃ for 24 to 48 h (Li et al., 2023a).

### Measurement of intracellular ferrous ion levels

Ferrous iron uptake kinetics were measured as follows: WT and AhΔ*feoB* strains were grown overnight (16 h, 37°C) in M9 minimal medium. A 2 ml aliquot was collected as the 0 min baseline sample, then FeSO_4_ was supplemented to a final concentration of 0.2 mM. Additional 2 ml culture samples were harvested at 5, 10, 15, 20, 25, 30, and 35 min post‑iron exposure and at least three independent biological replicates were performed. Bacteria were harvested by centrifugation, washed twice with iron-free PBS to eliminate extracellular iron, and lysed via sonication. After removing cell debris by centrifugation, clear supernatants were collected for intracellular ferrous ion measurement using the Ferrous Ion Content Assay Kit (Solarbio, BC5415), following the manufacturer’s instructions. At least three independent biological replicates were performed. A parallel Fe^2+^ standard curve was generated in each assay to calculate absolute Fe^2+^ concentrations of cell lysates (Fig. S6). Intracellular Fe^2+^ content was normalized to total protein quantified by the BCA kit (BCA standard curve, Fig. S7). Final Fe^2+^ levels were presented as pmol Fe^2+^ per mg total protein.

For determining intracellular ferrous ion levels, WT, AhΔ*feoB*, and CΔ*feoB* were cultivated in M9 minimal medium supplemented with 0.2 mM FeSO_4_. Bacteria were harvested at log-phase by centrifugation, washed with iron-free PBS and lysed via sonication. Supernatants were collected for intracellular ferrous ion measurement. At least three independent biological replicates were performed. Final intracellular Fe^2+^ levels were presented as nmol Fe^2+^ per mg total protein.

### Detergent sensitivity and oxidative stress assays

For sodium deoxycholate tolerance assays, strains were cultured in LB medium to an OD_600_ of approximately 0.9 and harvested by centrifugation. The cells were then washed twice with phosphate-buffered saline (PBS), resuspended in either 0.5% sodium deoxycholate or PBS and incubated at 37°C for 2 h, then serially diluted and plated on LB agar to determine viable cell counts. Survival rates were calculated as the ratio of CFUs obtained from treated groups to those from PBS controls (Li et al., 2022).

For oxidative stress resistance, 100 μl aliquots of bacterial cultures (OD_600_ ≈ 1.0) were evenly spread on the LB agar plates. Sterile filter paper disks containing 15 μl 1 M H_2_O_2_ were placed onto the agar surface. The inhibition zone diameters were measured after incubation to evaluate sensitivity to oxidative stress (Chen et al., 2023).

### Antibiotic susceptibility testing and determination of minimum inhibitory concentration (MIC)

Antibiotic susceptibility was assessed via Kirby–Bauer disk diffusion method using 25 antibiotics, including cefazolin, penicillin, ampicillin, novobiocin, sulfamethoxazole/trimethoprim, acetylspiramycin, vancomycin, teicoplanin, polymyxin B, streptomycin, gentamicin, amikacin, neomycin, ofloxacin, norfloxacin, levofloxacin, enrofloxacin, clindamycin, erythromycin, tetracycline, florfenicol, minocycline, doxycycline, lincomycin, and meropenem (Lo Sciuto et al., 2014; Matuschek et al., 2018). The clindamycin minimum inhibitory concentration (MIC) was further determined under normal and iron-limited (DIP-treated) conditions.

### Biofilm formation assay

Biofilm formation ability was quantified by crystal violet staining method in 24-well polystyrene plates (Liu et al., 2019). WT, AhΔ*feoB*, and CΔ*feoB* strains were prepared and harvested in LB medium, then washed with PBS. 500 µl of the bacterial suspension (OD_600_ = 0.2) was dispensed into 24-well plates, following static incubation at 30°C for 36 h. Sterile LB medium was used as a negative control. Non-adherent cells were eliminated by PBS washing. The remaining biofilms were fixed with 800 µl methanol for 30 min, followed by washing with distilled water and air-dried at 60°C for 1 h. 500 µl of 1% (w/v) crystal violet solution was added to each well and incubated for 15 min. Excess dye was removed by washing, followed by drying the plates at 60°C for 30 min. The stained biofilms were dissolved in 500 µl of 33% (v/v) glacial acetic acid, and absorbance was detected at 590 nm using a SpectraMax 190 microplate reader (Molecular Devices Corp., USA).

### Acridine orange staining

WT and AhΔ*feoB* overnight cultures were diluted to an OD_600_ of 0.2 in LB medium. A volume of 500 µl of the adjusted bacterial suspension was then added to 6-well polystyrene plates containing sterile glass coverslips. After incubation, the biofilms were gently washed with PBS, fixed with methanol, and air-dried. The samples were subsequently stained with a 100 μg/ml acridine orange in the dark for 30 min. Biofilms were examined using a Leica DM6 fluorescence microscope at 40× magnification. At least three images were captured from randomly selected fields on each coverslip (Conway et al., 2019).

### Transmission electron microscopy (TEM) observation

Transmission electron microscopy (TEM) analysis was performed as previously described (Seike et al., 2021) with minor modifications. Briefly, bacterial cells were collected by centrifugation at 3,000 × g for 5 min and subsequently washed twice with sodium cacodylate buffer. The suspension was then applied to carbon-coated copper grids, negatively stained with 2% (w/v) uranyl acetate and air-dried. Ultrastructural morphology was observed using a transmission electron microscope (H 600, Hitachi, Japan).

### RNA isolation, reverse transcription, and quantitative real-time PCR

Quantitative real-time PCR (qRT-PCR) analysis was performed to evaluate gene expression profiles as previously described (Gong et al., 2024). The transcription levels of *feoB*, five biofilm-associated genes, and 15 virulence-related genes were quantified by qRT-PCR with 16S rRNA as the internal reference. The primers are listed in Table S2. The biofilm-associated genes analyzed included methylthioadenosine/S-adenosylhomocysteine nucleosidase genes (*mtnN-1* and *mtnN-2*) and quorum-sensing-related genes (*luxR*, *ahyR*, and *ahyI*). The selected virulence-associated genes comprised flagellar and pilus-related genes (*flgE*, *flgL*, and *pilB*), a pilin subunit gene (*cblA*), type VI secretion system-related genes (*vasH* and *traA*), a type III secretion system gene (*ascV*), a serine peptidase gene (*degQ*), toxin-related genes (*aerA* and *hlyA*), outer membrane protein genes (*ompA* and *ompTS*), a two-component regulatory gene (*ompR*), a lipopolysaccharide biosynthesis gene (*rfaF*), and 4-amino-4-deoxychorismate lyase (ADCL).

Total RNA was extracted and its concentration was quantified before cDNA synthesis. First-strand cDNA was generated using the FastQuant RT Kit (with gDNase) (Tiangen, China). Quantitative PCR was conducted using SYBR Green I on an ABI 7500 Real-Time PCR system (Applied Biosystems, USA). Each reaction was performed in a final volume of 10 μl under the following cycling conditions: initial denaturation at 95°C for 20 s, followed by 45 cycles of 95°C for 20 s, 55°C for 20 s, and 72°C for 20 s (Chen et al., 2025). Relative mRNA expression was calculated using ABI 7500 software (version 2.0.6). Each experiment was conducted with five independent replicates. The 16S rRNA was used as the reference housekeeping gene, and no-template negative controls were run in parallel. The expression stability of 16S rRNA in different strains were analyzed using RefFinder, an online tool for the comprehensive evaluation of RGs. RefFinder integrates four widely used algorithms—ΔCt, geNorm, NormFinder, and BestKeeper to assess the stability of reference genes (Xie et al., 2023).

Gene expression levels were normalized to the 16S rRNA via the 2^-ΔΔCT^ method. Primer amplification efficiencies were determined by standard curve analysis, with all efficiencies ranging from 90% to 110% (Bustin et al., 2009).

### Determination of AhΔ*feoB* virulence in a mouse model

Female BALB/c mice (4–6 weeks old) were used to evaluate bacterial virulence. The bacteria were cultured in LB medium at 37°C to the log phase, then centrifuged, washed twice with sterile saline, and adjusted to the desired concentration based on OD_600_ values. Mice were grouped into six groups of five and intraperitoneally injected with 1.0 ml of bacterial suspension containing 0.5 × 10^7^ to 2.5 × 10^7^ CFU/ml prepared in sterile Dulbecco’s phosphate-buffered saline (DPBS). Survival rates were monitored daily for 14 days (Liu et al., 2019). The virulence of the WT and AhΔ*feoB* strains was assessed by determining the 50% lethal dose (LD_50_).

### Protein sample preparation

Bacterial cultures were inoculated into 10 ml of LB medium at a 1:100 (v/v) ratio and incubated at 30°C until the OD_600_ reached approximately 1.0. Cells were harvested and washed three times with PBS. The cell pellets were then resuspended in 0.1 ml lysis buffer containing 8 M urea in 100 mM Tris–HCl (pH 8.5). Cell disruption was achieved through repeated freeze–thaw cycles followed by sonication (30 s pulses at 20% amplitude for five cycles). The lysates were subsequently centrifuged at 18,000 × g for 15 min at 4°C, and the supernatants were collected for further analysis. Protein concentration was determined using the Bradford method (Reddy et al., 2024). For proteomic analysis, proteins were reduced with 10 mM dithiothreitol at 37°C for 30 min, alkylated with 20 mM iodoacetamide at 25°C for 45 min in the dark and digested with trypsin at a ratio of 1:100 (w/w) at 37°C for 12 h. Peptides were desalted using a C18 solid-phase extraction column (CNW, Anpel, China). Subsequently, peptide concentrations were normalized using a BCA Protein Assay Kit (Pierce, USA) and qualified by liquid chromatography–mass spectrometry (LC–MS; Thermo Fisher Scientific, USA) prior to proteomic analysis (Agwunobi et al., 2022).

### Data-independent acquisition (DIA) proteomics analysis

Data-independent acquisition (DIA) proteomics was performed on a high-resolution mass spectrometry platform (Wang et al., 2022). Protein samples were reconstituted in solvent A (99.9% H_2_O, 0.1% formic acid) supplemented with indexed retention time (iRT) peptides (Biognosys, Switzerland). DIA-based quantitative analysis was performed on a Vanquish^TM^ Neo UHPLC system coupled to an Orbitrap Exploris 480 mass spectrometer (Thermo Fisher Scientific, USA).

Samples were initially loaded onto a C18 reverse-phase trap column (5 μm particle size, 300 μm inner diameter × 5 mm length; PEPMAP NEO, Thermo Fisher) and subsequently separated on a C18 analytical column (1.5 μm particle size, 75 μm inner diameter × 250 mm length; Waters, USA) at a flow rate of 300 nl/min. Peptides were ionized via electrospray in positive ion mode at 2,300 V and analyzed under DIA acquisition settings.

Raw mass spectrometry data were processed using Spectronaut software (version 18.0; Biognosys, Switzerland) with default parameters, and a false discovery rate (FDR) threshold of < 1%. Protein identification was based on the *A. hydrophila* ATCC 7966 protein database derived from transcriptomic sequencing (NCBI accession: NC_008570.1).

Differentially expressed proteins (DEPs) were defined as those exhibiting a fold change (FC) > 1 or < 0.667 and a *P* value of < 0.05. Functional annotation and enrichment analyses were conducted using the Majorbio Cloud platform (https://cloud.majorbio.com). All proteomic data are provided in Table S1.

### Statistical analysis

All statistical analyses were conducted using GraphPad Prism 8.0. Differences between two groups were analyzed using Student’s t-test, while one-way analysis of variance (ANOVA) was applied for multiple-group comparisons. Survival data and LD_50_ values were analyzed via the log-rank test. Statistical significance was defined as *P < 0.05, **P < 0.01, and ***P < 0.001.

## Results

### Effects of *feoB* mutation on the hemolytic and motility activity of *A. hydrophila*

The qRT-PCR results confirmed that the *feoB* gene was not expressed in AhΔ*feoB*, but was expressed in both the wild-type (WT) and complemented strains (CΔ*feoB*). It showed that AhΔ*feoB* and CΔ*feoB* were successfully constructed (Fig. 1A). Hemolysin is an important virulence factor in many aquatic bacteria. All strains displayed equal α-hemolytic activity on sheep blood agar (Fig. 1B). The swimming diameter of the WT was approximately twice that of AhΔ*feoB* (27 mm). However, the swarming capacity of AhΔ*feoB* showed no significant difference compared with WT. The motility phenotype was restored in the CΔ*feoB* strains (Figs. 1C and S1).

### The *feoB* gene does not affect the growth of *A. hydrophila*

In standard LB medium, the WT strain showed a typical growth curve, with a short lag phase (0–2 h), followed by a log phase during which major bacterial growth occurred (2–15 h) and then a stationary phase (15–40 h). The AhΔ*feoB* showed a slightly reduced growth rate during the exponential phase but ultimately reached CFU levels comparable to those of the WT strain at the stationary phase. The growth rate of the complemented strain CΔ*feoB* was consistent with that of the WT. In LB medium supplemented with 0.6 mM 2,2'-dipyridyl (DIP), the growth of all strains was inhibited, with peak OD_600_ values reduced to approximately 0.5. No significant differences were observed in growth curves (Fig. 2A and 2B).

In M9 minimal medium, all strains exhibited lower peak OD_600_ values and entered a gradual decline in the late growth phase after 20 h (Fig. 2C). Supplementation of exogenous iron (0.2 mM FeSO_4_) restored consistent growth profiles across the three strains with peak OD_600_ of 1.0 and no evident decline in the stationary phase (Fig. 2C and 2D).

### Deletion of *feoB* affects ferrous iron transport in *A. hydrophila*

To investigate the function of *feoB* in ferrous iron acquisition of *A. hydrophila*, Fe^2+^‑uptake kinetics were determined for WT and AhΔ*feoB* by Michaelis‑Menten fitting (Fig. 3). The WT exhibited a *V*_max_ of 299 nmol Fe^2+^ mg^-1^ protein and *K*_m_ of 10.26, whereas the AhΔ*feoB* exhibited a reduced *V*_max_ of 237 nmol Fe^2+^ mg^-1^ protein and elevated *K*_m_ of 15.9. These data indicate that deletion of *feoB* diminished both the maximal transport capacity and substrate affinity for Fe^2+^. Nevertheless, substantial Fe^2+^ uptake was still detectable in AhΔ*feoB*, implying the existence of additional ferrous‑iron acquisition systems.

The WT had an intracellular ferrous iron concentration of 62.1 nmol, significantly elevated relative to AhΔ*feoB* (43.1 nmol) at log-phase. The CΔ*feoB* recovered ferrous iron accumulation comparable to WT (Fig. 4A). These findings verify that *feoB* fulfills an important role in physiological ferrous iron uptake by *A. hydrophila*.

### Deletion of *feoB* affects envelope integrity in *A. hydrophila*

Bacterial sensitivity to oxidative stress and membrane-disrupting agents was evaluated using hydrogen peroxide and sodium deoxycholate assays. The inhibition zone diameter of AhΔ*feoB* under H_2_O_2_ oxidative stress was larger than that of the WT strain and the phenotype was restored in the CΔ*feoB* strains (Fig. 4B). These results indicated that AhΔ*feoB* was more sensitive to oxidative stress. Ionic detergents, such as sodium deoxycholate, are effective in solubilizing both cytoplasmic and nuclear cellular membranes. The results showed that AhΔ*feoB* was more susceptible to 0.5% sodium deoxycholate than the WT strain (Figs. 4C and S2). These results indicate that *feoB* is required to maintain membrane integrity and envelope integrity in *A. hydrophila*.

### Role of *feoB* on antibiotic sensitivity of *A. hydrophila*

Compared to WT, AhΔ*feoB* mutant exhibited increased sensitivity to several antibiotics. AhΔ*feoB* susceptibility to gentamicin shifted from Resistant (R) to susceptible (S), while susceptibility to streptomycin and neomycin changed from R to intermediate (I); susceptibility to enrofloxacin was altered from intermediate (I) to susceptible (S). Notably, the AhΔ*feoB* mutant exhibited a 15 mm increase in the clindamycin inhibition zone (Table 2). The MIC assay showed that deletion of the *feoB* gene increased sensitivity to clindamycin, alongside reduced MIC from 0.78 mg/ml to 0.39 mg/ml under both iron-replete and DIP-induced iron-limited conditions (Fig. 4D). Although the response was categorized as resistant based on the clinical breakpoint (≤ 19 mm for resistant), this marked phenotypic improvement suggested that impaired ferrous transport likely increases membrane permeability and weakens stress tolerance, restoring partial clindamycin susceptibility.

### Deletion of *feoB* affects biofilm formation

The results indicated that AhΔ*feoB* formed significantly less biofilm than the WT, suggesting an overall positive effect of *feoB* on biofilm formation. The biofilm formed by CΔ*feoB* restored the parental phenotype (Figs. 5A and S3). The effect of *feoB* gene deletion on the expression levels of biofilm-associated genes were significant downregulation of *mtnN-1*, *mtnN-2*, *luxR*, *ahyR*, and *ahyI* in AhΔ*feoB* (Fig. 5B). As observed under a fluorescence microscope, the WT strain formed a thick, dense, well-organized biofilm on the glass surface. In contrast, the AhΔ*feoB* mutant formed a much thinner, loosely organized biofilm, indicating that deletion of *feoB* significantly inhibited biofilm formation (Fig. 5C).

### Morphological characterization of *A. hydrophila*

The effect of *feoB* deletion on the cellular morphology of *A. hydrophila* was examined by TEM, which revealed that WT cells were rod-shaped, tightly packed, and uniform in morphology. Though AhΔ*feoB* cells retained normal morphology, cells aggregated more loosely and displayed drastically fewer incomplete flagella. Additionally, pili density was markedly decreased with irregular distribution. Numerous outer membrane vesicles of variable sizes were clearly observed around the outer membrane of AhΔ*feoB* cells. Many of these vesicles appeared to bud or pinch off from the cell surface, often connected to the outer membrane by thin filamentous structures. The morphology of CΔ*feoB* restored the parental phenotype (Fig. 6). Collectively, *feoB* deletion disrupts normal structure of the bacterial cell envelope.

### Quantitative real-time PCR analysis

Stability analysis of 16S rRNA in different strains were analyzed using RefFinder. ΔCt analysis showed that 16S rRNA was most stable in AhΔ*feoB* (SD = 0.53), followed by WT (SD = 0.68) and CΔ*feoB* (SD = 0.80). BestKeeper ranked stability as CΔ*feoB* (0.3) > AhΔ*feoB* (0.35) > WT (0.48). GeNorm gave the lowest M‑value (0.41) for WT and AhΔ*feoB*, and the highest M‑value (0.67) for CΔ*feoB*. NormFinder offer the ranking: AhΔ*feoB* (0.2) > WT (0.58) > CΔ*feoB* (0.76). Collectively, 16S rRNA was stably expressed among tested strains and qualified as a qRT‑PCR reference gene.

The transcriptional levels of 15 virulence-associated genes were detected. In AhΔ*feoB*, 12 genes (*aerA*, *degQ*, *ompA*, *flgE*, *ompTS*, *hlyA*, *rfaF*, *traA*, *cblA*, *acuC*, and *ascV*) were significantly downregulated. By contrast, the expression of *vasH*, *pilB* and *flgL* were significantly upregulated. There is no significant difference between the WT and CΔ*feoB* regarding the expression of these genes (Fig. 7A).

### Pathogenesis in the BALB/c mouse model

The 14-day mouse infection assay showed similar mortality and lethal dose (LD_50_) between WT (0.77 × 10^6^ CFU/ml) and AhΔ*feoB* (0.72 × 10^6^ CFU/ml), indicating that *feoB* deletion did not alter the lethal pathogenicity of *A. hydrophila* (Fig. 7B).

### Data-independent acquisition (DIA)-based proteomics result

The mass spectrometry proteomics data have been deposited to the ProteomeXchange Consortium (https://proteomecentral.proteomexchange.org) via the iProX partner repository with the dataset identifier IPX0018386000. Protein intensities across three biological replicates showed high reproducibility (Pearson's r > 0.9), suggesting the reliability of the proteomics data (Fig. 8A). Principal component analysis (PCA) analysis revealed distinct proteomic profiles between WT and the *feoB* mutant with PC1 accounted for 53.5% of total variance, and PC2 accounted for 13.70%. Volcano plot identified 435 differentially expressed proteins (DEPs) (FC > 1 and p-value < 0.05) in AhΔ*feoB*, including 226 upregulated and 209 downregulated proteins (Figs. 8B, 8C, and S4).

### Cluster analysis of DEPs

The DEPs were categorized into ten expression clusters. The most significant pathway was cluster C9 including arginine deiminase and catabolic processes, oxidoreduction−driven active transmembrane transporter activity, structural constituent of chromatin, proton and inorganic molecular transmembrane transporter activity and glutamine family amino acid catabolic process. The enrichment analysis results were all statistically significant (*P* < 0.001) (Fig. S5).

### GO enrichment analysis of DEPs

The DEPs between WT and the *feoB* group were categorized into three main domains: biological process (BP), molecular function (MF), and cellular component (CC). In the BP pathway, amino acid catabolism was significantly enriched, indicating that amino acid catabolism served as the core function following *feoB* deletion. Terms such as carboxylic acid catabolism, organic acid catabolism, and cellular catabolism were also highly enriched, demonstrating a global enhancement of catabolism. In the MF pathway, the terms of structural constituents of chromatin and structural molecule activity were significantly enriched, suggesting important changes in chromatin structure and cytoskeleton. In the CC pathway, the term of bacterial flagellum was significantly enriched, indicating upregulation of flagellar structure-related proteins, which may be associated with bacterial changes in motility and biofilm formation (Fig. 9A).

The downregulated proteins were shown in Fig. 9B. In the BP pathway, terms related to metal ion (especially ferrous ion) transmembrane transport were significantly enriched and suppressed. Meanwhile, terms such as maintenance of location in cell, sequestering of metal ion, intracellular sequestering of iron ion, and sequestering of iron were also highly enriched. This result is directly related to the function of *feoB* as a ferrous iron transporter, indicating metal ion transport and intracellular localization constitute the core function of *feoB*. In the MF pathway, protein catalytic activity and inorganic ion transmembrane transport were significantly weakened. Additionally, enzyme activity-related terms were enriched, indicating that nucleic acid metabolism and cell wall remodeling processes were also downregulated. In the CC pathway, the terms of membrane and TAM protein secretion complex were significantly enriched, indicating that the expression of membrane-related components and the protein secretion system were generally repressed. These terms were directly related to the membrane transport functions.

### KEGG enrichment analysis of DEPs

KEGG metabolic pathway enrichment analysis revealed metabolic (M) pathways were predominantly enriched, with amino acid and energy metabolism emerging as core upregulated functions. In particular, the arginine and proline metabolism pathway was the most highly enriched pathway containing the largest number of DEPs. This result confirmed the central role of arginine metabolism in the strain's adaptation to iron-deficient environments. Secondary metabolite biosynthesis showed the highest enrichment factor, activated to counter iron-limited stress. Other metabolic pathways were also significantly enriched, suggesting systematic reconstruction of the strain's global metabolic network, particularly amino acid and energy metabolism. The Cellular Processes (CP) pathway including bacterial motility, signal transduction and flagellar assembly were also enhanced, representing an adaptive strategy for nutrient acquisition in iron-deficient environments. The upregulation of NOD-like receptor and stress response signaling suggested activation of innate immunity and response to cellular stress induced by iron deficiency (Fig. 9C).

As shown in Fig. 9D, in the M pathway, amino acid and carbohydrate metabolism were significantly downregulated, indicating polysaccharide degradation and carbohydrate metabolism processes were strongly inhibited. Contrasting activated amino acid catabolism, nitrogen metabolism and amino acid synthesis declined, indicated that the core energy production and carbon metabolism networks underwent global reconstruction. In the CP pathway, suppressed chemotaxis weakened chemotactic and motion-sensing capabilities. The inhibited lipopolysaccharide biosynthesis compromised membrane integrity and environmental fitness. The homologous recombination was downregulated, suggesting that DNA recombination and repair processes were inhibited, potentially affecting the strain's genomic stability. In Organismal Systems (OS), repressed cobalamin (vitamin B12) transport and metabolism pathway correlated with impaired metal ion trafficking.

### Circular enrichment analysis of DEPs

The circular enrichment plot categorized DEPs into three functional groups: Metabolism, Cellular Processes, and Organismal Systems. The metabolic-related classifications dominated the DEP distribution, with arginine and proline metabolism (aha00330, 49 proteins) and alanine, aspartate and glutamate metabolism (aha00250, 39 proteins) as the most enriched modules, indicating metabolism as the core regulatory pathway. Several metabolic-related pathways exhibited extremely high enrichment significance (*P* < 0.01), suggesting profound metabolic perturbations in the AhΔ*feoB*.

Protein expression patterns differed markedly across functional categories: metabolic pathways were predominantly upregulated, while cellular processes including membrane transport and flagellar assembly were mostly downregulated. This expression pattern reflects an adaptive strategy of enhanced catabolism and repressed membrane transport under iron deficiency, which remodels bacterial resource allocation. The metabolic pathways showed higher Rich Factor values than other categories, further verifying that metabolic reprogramming serves as the central mechanism for AhΔ*feoB* to adapt to iron-limited conditions (Fig. 10A).

### GSEA analysis of DEPs

Unlike GO/KEGG enrichment analysis that focuses on individual DEPs, Gene Set Enrichment Analysis (GSEA) can capture subtle but coordinated changes. GSEA results indicated that seven pathways were significantly upregulated in AhΔ*feoB*: Folate biosynthesis, Flagellar assembly, Quorum sensing, ABC transporters, Amino acid biosynthesis, Bacterial secretion system and Ribosome. Folate biosynthesis pathway was the most activated pathway, facilitating DNA synthesis, cell proliferation and biosynthesis. Activation of flagellar assembly and quorum-sensing pathways improves motility, group behavior and virulence. The activation of ABC transporters, secretion system and ribosomals enhance substance transport and protein synthesis. By contrast, thirteen central carbon metabolism pathways were repressed, revealing global inhibition of energy metabolism (Fig. 10B).

Overall, the *feoB* deletion reshapes the global regulatory network of *A. hydrophila*. Upregulated biosynthesis, motility, virulence and translation pathways maintain growth and adaptability, whereas suppressed energy metabolism reduces energy expenditure and remodels metabolism. This dual regulation enables *A. hydrophila* to survive disrupted under disrupted iron homeostasis.

## Discussion

Iron is essential for the survival and metabolism of pathogenic bacteria, and iron homeostasis is strongly associated with bacterial virulence, environmental adaptation, and antimicrobial resistance. In Gram-negative bacteria, the Feo system is a major pathway for ferrous iron uptake and its component gene *feoB* is widely recognized as critical for iron acquisition and pathogenicity in many bacteria (Guan et al., 2025; Huang et al., 2023; Kammler et al., 1993; Naikare et al., 2006; Tsolis et al., 1996).

In this study, the *feoB* gene of *A. hydrophila* ATCC 7966 was successfully deleted via homologous recombination, generating AhΔ*feoB* mutant and its complemented strain. We further explored the biological role of *feoB* and global proteomic differences between the mutant and the wild-type (WT) strains. The results demonstrated that *feoB* participates in multiple regulatory processes in *A. hydrophila*, with functions different from those reported in other bacteria.

Iron acts as an essential cofactor for enzymatic reactions, DNA synthesis and repair, electron transport, oxidative phosphorylation, and various metabolic pathways (Strzelecki and Nowicki, 2025). Nevertheless, the growth rate of AhΔ*feoB* was consistent with that of the WT under iron-restricted conditions. This finding is consistent with previously reported studies on *Campylobacter jejuni* and *Aeromonas veronii* (Guan et al., 2025; Naikare et al., 2006), but contrasted with reports in *Escherichia coli*, *Salmonella enterica*, and *Riemerella anatipestifer*, in which *feoB* disruption leads to severe growth defects under iron limitation (Huang et al., 2023; Kammler et al., 1993; Tsolis et al., 1996).

Bacterial motility plays an essential role in survival, environmental adaptation, and pathogenicity. Motility assays showed that *feoB* deletion significantly inhibited swimming motility without obvious influence on swarming motility. Transmission electron microscopy revealed decreased flagellar density and structural abnormalities in the mutant, indicating that *feoB*-dependent iron-uptake supports normal flagellar assembly and motility. Similar results have been observed in *A. veronii*, where the Δ*feoB* mutant exhibited impaired swimming ability, severe disruption of flagellar structure, and flagellar breakage, accompanied by downregulation of flagellar biosynthesis-related genes (Guan et al., 2025). Ferrous iron also serves as a regulatory signal to stimulate flagellar gene transcription and swimming motility in *Stenotrophomonas maltophilia* (Zhang et al., 2022). These data suggest that iron availability is required not only for the flagellar components synthesis but also to provide energy for motility.

The AhΔ*feoB* displayed increased susceptibility to H_2_O_2_-induced oxidative stress and sodium deoxycholate-mediated membrane damage, reflecting weakened cellular defense capacity. Similar phenotypes have been documented in other bacterial species (Guan et al., 2025; Wang et al., 2021). Conversely, *feoB* deletion decreased H_2_O_2_ susceptibility in *R. anatipestifer*, suggesting that iron-dependent stress responses are species-specific (Huang et al., 2023). Iron is an essential cofactor for antioxidant enzymes such as superoxide dismutase (SOD) and catalase. Consequently, disturbed intracellular iron balance reduces enzyme activity and raises oxidative stress susceptibility (Liu et al., 2025a; Nandi et al., 2019). TEM observation detected abundant outer membrane vesicle-like structures, reduced flagella and pili density and loosely arranged cells in AhΔ*feoB* cells. These structural changes indicated disruption of the cell envelope integrity, higher membrane permeability to external stressors. Alterations in membrane integrity may interfere with flagella and pili assembly, thereby further contributing to the enhanced mutant’s stress sensitivity.

Antibiotic susceptibility testing showed that *feoB* deletion moderately elevated bacterial sensitivity, confirming the linkage between bacterial iron transport systems and antimicrobial resistance. Aminoglycosides including streptomycin, gentamicin, and neomycin are widely used in aquaculture to control bacterial infections, whereas enrofloxacin is commonly used to treat *A. hydrophila*-associated diseases (Wainwright, 1991; Zhang et al., 2024). The bactericidal efficacy of these antibiotics depends largely on active cellular metabolism, disrupted iron homeostasis disturbs energy production and protein synthesis, alter membrane permeability and consequently influencing antibiotic susceptibility (Guan et al., 2025; Pan et al., 2023; Zhu et al., 2017). Although AhΔ*feoB* remained clinically resistant to clindamycin, the reduction in MIC indicates partial susceptibility increase. Clindamycin mainly targets Gram-positive bacteria and anaerobes with limited efficacy against most Gram-negative organisms, including *Aeromonas* spp. (Zdanowicz et al., 2020). Two reasons may explain this change: firstly, compromised membrane integrity facilitates antibiotic penetration and intracellular accumulation; secondly, disruption of iron homeostasis may impair stress response pathways and metabolic capacity, lowering bacterial tolerance to antibiotics.

Biofilm formation is a vital virulence trait of *A. hydrophila*, facilitating adhesion, colonization and stress resistance (Zhao et al., 2022). Our study found that *feoB* deletion markedly suppressed biofilm formation, AhΔ*feoB* formed thinner, less structured biofilms compared with the WT strain. Consistently, *feoB* disruption also inhibits biofilm development in *A. veronii* and *Legionella pneumophila* (Guan et al., 2025; Lopez et al., 2023), implying a conserved correlation between iron acquisition and biofilm formation across bacterial species (Oliveira et al., 2021). Iron availability is a key determinant of biofilm development and biofilm maturation is severely disrupted upon *feoB* deletion.

Correspondingly, multiple biofilm-associated genes were significantly downregulated in AhΔ*feoB*. These genes participate in quorum sensing, nucleoside metabolism, and other pathways that are essential for biofilm initiation, maturation, and structural maintenance (Kirke et al., 2004; Mao et al., 2020; Silva et al., 2015). Their decreased expression directly contributes to defective biofilm formation. Meanwhile, reduced flagellar density and altered membrane structure in the mutant further hinder biofilm development, as both motility and membrane integrity are essential for initial surface attachment and biofilm architecture. Decreased biofilm formation is also associated with increased antibiotic susceptibility, such as elevated neomycin sensitivity (Zhang and Lu, 2003), consistent with the antibiotic susceptibility results. These results confirm the conserved regulation of biofilm formation by *feoB* within the genus *Aeromonas*, and targeting *feoB* could retain biofilm formation and improve antimicrobial efficacy against *A. hydrophila*.

In numerous bacterial species, *feoB* deletion reduces colonization capacity or attenuate virulence (Fetherston et al., 2012; Huang et al., 2023; Runci et al., 2019; Sabri et al., 2008; Stojiljkovic et al., 1993; Tsolis et al., 1996; Velayudhan et al., 2000; Wyckoff et al., 2006). However, our study revealed comparable LD_50_ values between the AhΔ*feoB* and WT in the BALB/c mouse infection model. These results demonstrated that *feoB* deletion did not significantly weaken the virulence of *A. hydrophila*. Multiple compensatory mechanisms account for the unchanged virulence of AhΔ*feoB*. Despite defective biofilm formation and abnormal membrane structure, hemolytic activity and swarming motility (two virulence factors associated with host cell damage and tissue invasion) remained unaltered. Moreover, DIA-based proteomics identified several activated pathogenic pathways including flagellar assembly and quorum sensing, which represent a compensatory molecular upregulation triggered by genetic deficiency. Pathway enrichment at the proteome level reflects molecular transcriptional/translational adaptive responses, rather than directly equating to final phenotypic performance. Although related pathway proteins were enriched, the compensatory upregulation was insufficient to restore the actual physiological functions. Multiple downstream functional cascades and post-translational modifications may still be impaired, ultimately leading to the reduced swimming motility and biofilm formation phenotypes we observed. As reported, under iron-limitation, *A. hydrophila* upregulates multiple virulence-related traits, including protease, hemolysin, and lipase activities as well as motility, ultimately increasing host mortality (Teng et al., 2018). These findings demonstrate that virulence of *A. hydrophila* is controlled by a sophisticated coordinated regulatory network. Accordingly, single deletion of *feoB* is insufficient to markedly attenuate its pathogenicity, suggesting therapies targeting *feoB* should be combined with other virulence-related targets to achieve better control of infection (Feng et al., 2016; Raphael and Joens, 2003).

Iron is indispensable for diverse bacterial cellular processes, functioning as heme or iron-sulfur cluster cofactors in proteins involved in electron transport, oxidative stress sensing, and energy production (Lau et al., 2016). In this study, DIA-based proteomics uncovered the global regulatory effects of *feoB* on the proteome of *A. hydrophila*. GO and KEGG functional enrichment analyses showed that upregulated proteins were mainly enriched in amino acid catabolic pathways and secondary metabolite biosynthesis, whereas downregulated proteins were primarily related to metal ion (especially iron) transport, and membrane-related functions. These patterns resemble adaptive responses to iron starvation in *Pseudomonas aeruginosa*. Iron-limited proteomic studies in *P. aeruginosa* showed enhanced amino acid catabolism, remodeled energy metabolism via iron-independent entry of carbon into the tricarboxylic acid (TCA) cycle, increased abundance of twitching motility-related proteins, and repressed amino acid biosynthesis (Nelson et al., 2019). Upregulated amino acid catabolism in AhΔ*feoB* represents an adaptive strategy to generate energy and metabolic intermediates under disrupted iron homeostasis. The reduced ion transport proteins directly reflects impaired *feoB*-mediated iron uptake, and decreased membrane-associated proteins match the structural defects of AhΔ*feoB*.

GSEA further indicated the metabolic reprogramming prioritizes essential biosynthesis and regulatory pathways while downregulating energy-intensive central carbon metabolism, allowing *A. hydrophila* to maintain basic growth and environmental adaptability with defective ferrous iron uptake. A similar regulatory pattern has been reported in iron-limited *A. hydrophila* NJ-35. GO analysis revealed tight regulation of metabolism, biosynthesis, transmembrane transport, redox homeostasis and quorum sensing, while KEGG analysis showed upregulated quorum sensing and downregulated carbon metabolism pathways (Hood and Skaar, 2012).

## Conclusion

This study systematically characterizes biological functions of the ferrous iron transporter FeoB in *Aeromonas hydrophila* ATCC 7966. The results indicated that *feoB* is critical for maintaining membrane integrity, modulating stress responses, altering antibiotic susceptibility, regulating biofilm development, and remodeling global protein expression patterns. Deletion of *feoB* causes prominent morphological changes, enhanced sensitivity to oxidative stress and membrane-damaging agents, inhibited biofilm formation and downregulated multiple biofilm-associated genes. Despite its central role in iron transport, *feoB* deletion neither impair bacterial growth under iron limitation nor attenuates virulence in the BALB/c mouse model. Proteomic data revealed extensive metabolic reprogramming after *feoB* deletion, including enhanced amino acid catabolism and repressed metal-ion transport and membrane-related processes, further highlighting the central role of *feoB* in coordinating iron homeostasis and bacterial environmental adaptation.

These results deepen our understanding of the interplay between iron uptake and environmental adaptation in *A. hydrophila* and identify *feoB* as a potential antimicrobial target. Nevertheless, bacterial growth and pathogenicity remained unimpaired. This indicates that targeting *feoB* alone cannot achieve optimal therapeutic efficacy; combinatorial strategies against multiple regulatory pathways are therefore required to realize effective antibacterial outcomes.

## Figures and Tables

**Fig. 1.**
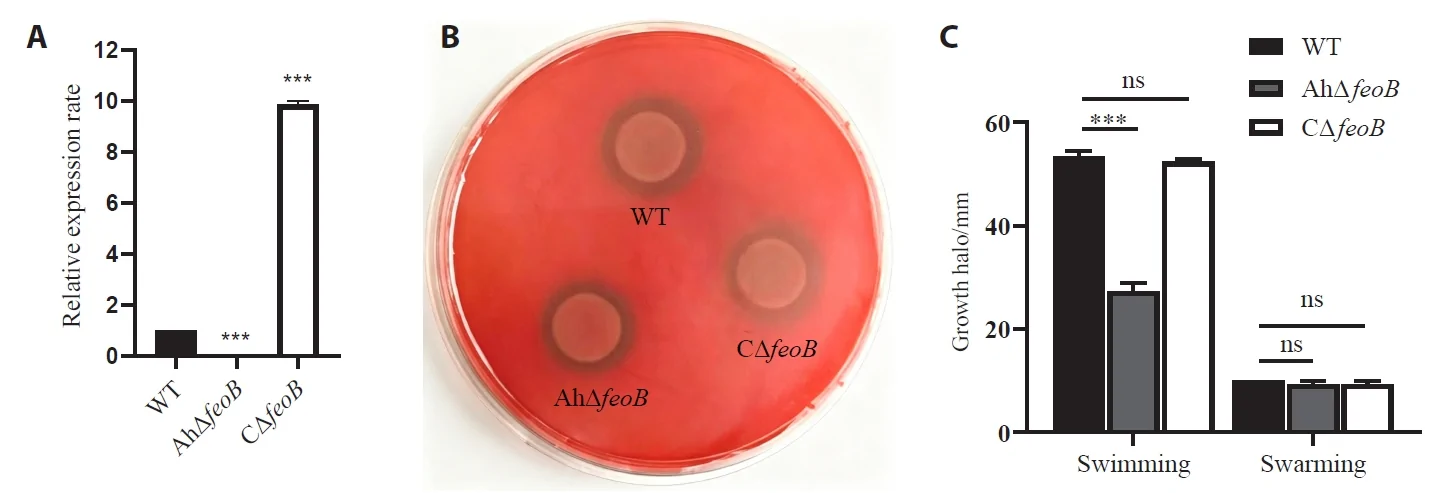
*feoB* gene expression, hemolytic activity and motility assay of WT, AhΔ*feoB* and CΔ*feoB*. (A) Transcript level of *feoB*, (B) hemolytic activity, and (C) motility.

**Fig. 2.**
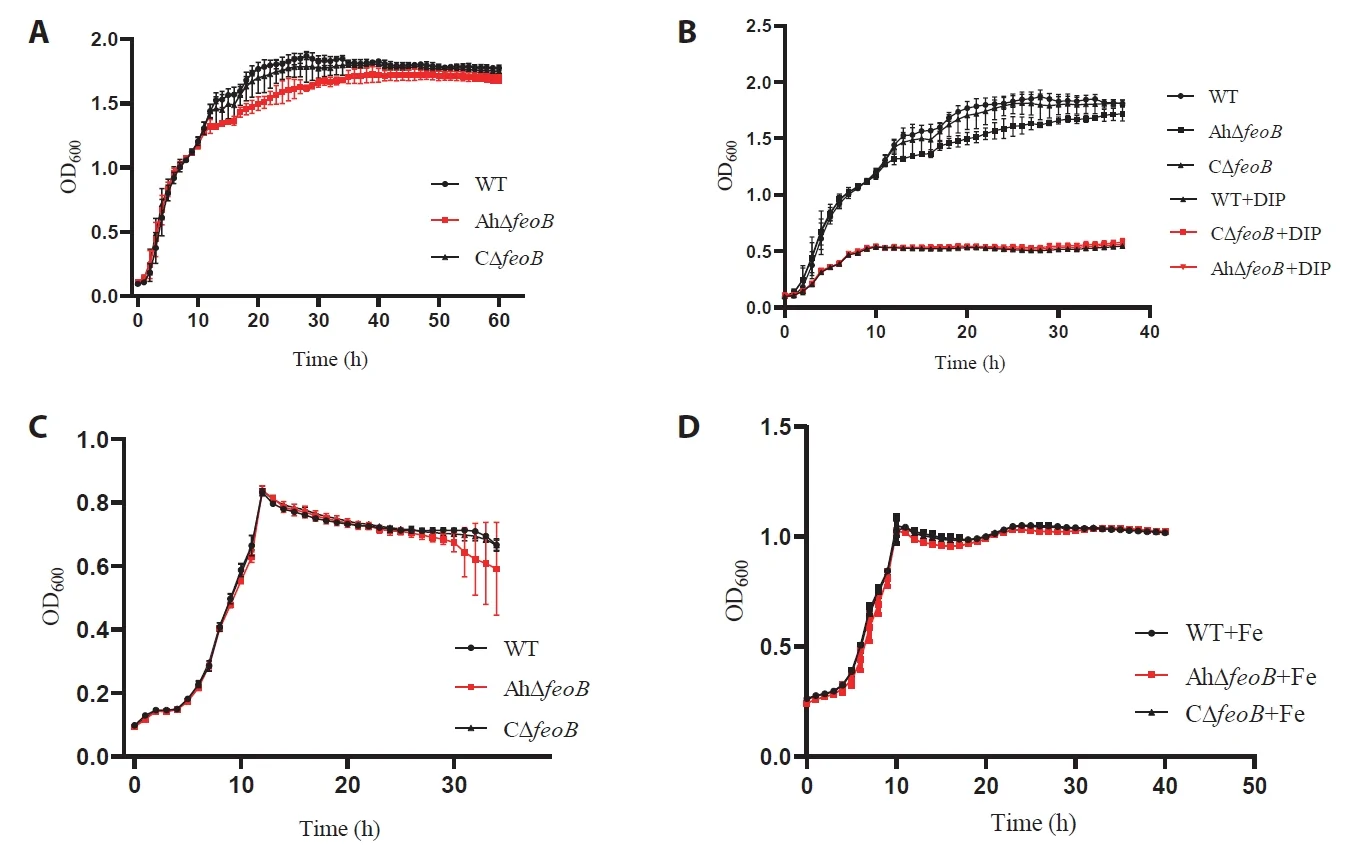
Growth characteristics of WT and AhΔ*feoB* and CΔ*feoB*. (A, B) Growth curves in LB with or without 2,2'-dipyridyl (DIP). (C, D) Growth curve in M9 with or without 0.2 mM FeSO_4_ supplementation.

**Fig. 3.**
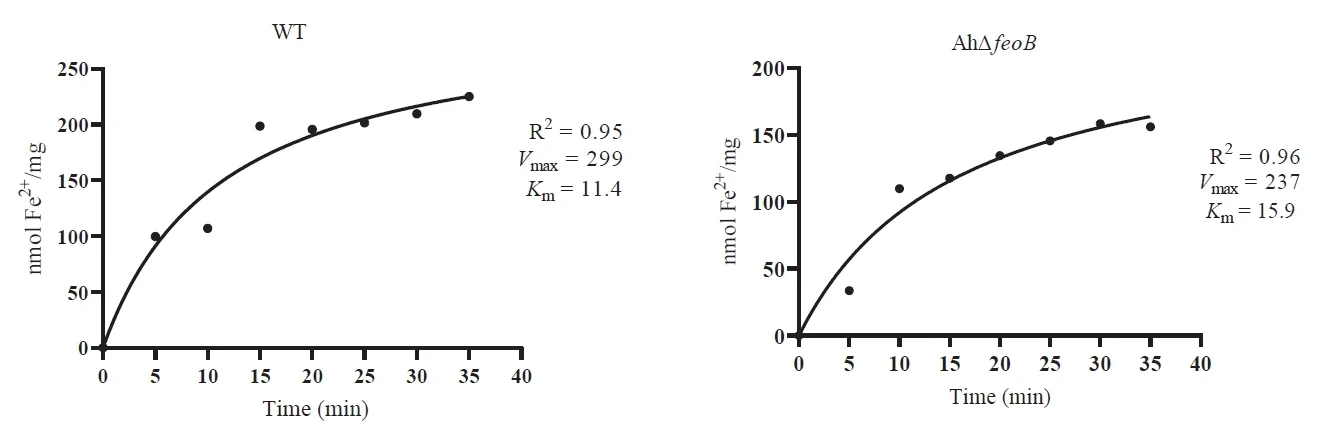
Kinetics of Fe^2+^ uptake in WT and AhΔ*feoB*. Cellular Fe^2+^ uptake was measured over 35 min incubation and fitted with the Michaelis‑Menten model. Kinetic parameters (R^2^, *V*_max_, and *K*_m_) values are shown for each strain.

**Fig. 4.**
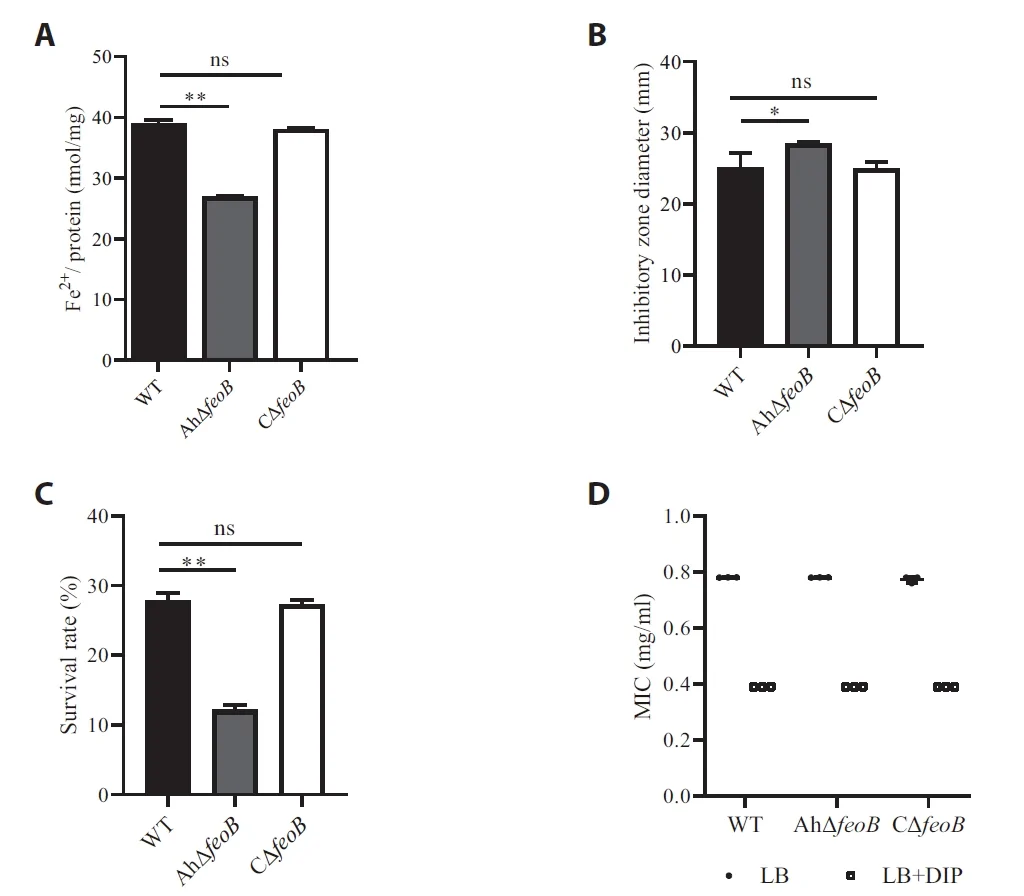
Intracellular ferrous ion levels, stress tolerance and MIC of clindamycin against WT and AhΔ*feoB*. (A) Intracellular ferrous ion levels. (B) Oxidative stress sensitivity. (C) Survival rates under 0.5% sodium deoxycholate treatment. (D) MIC determination of clindamycin with or without DIP exposure.

**Fig. 5.**
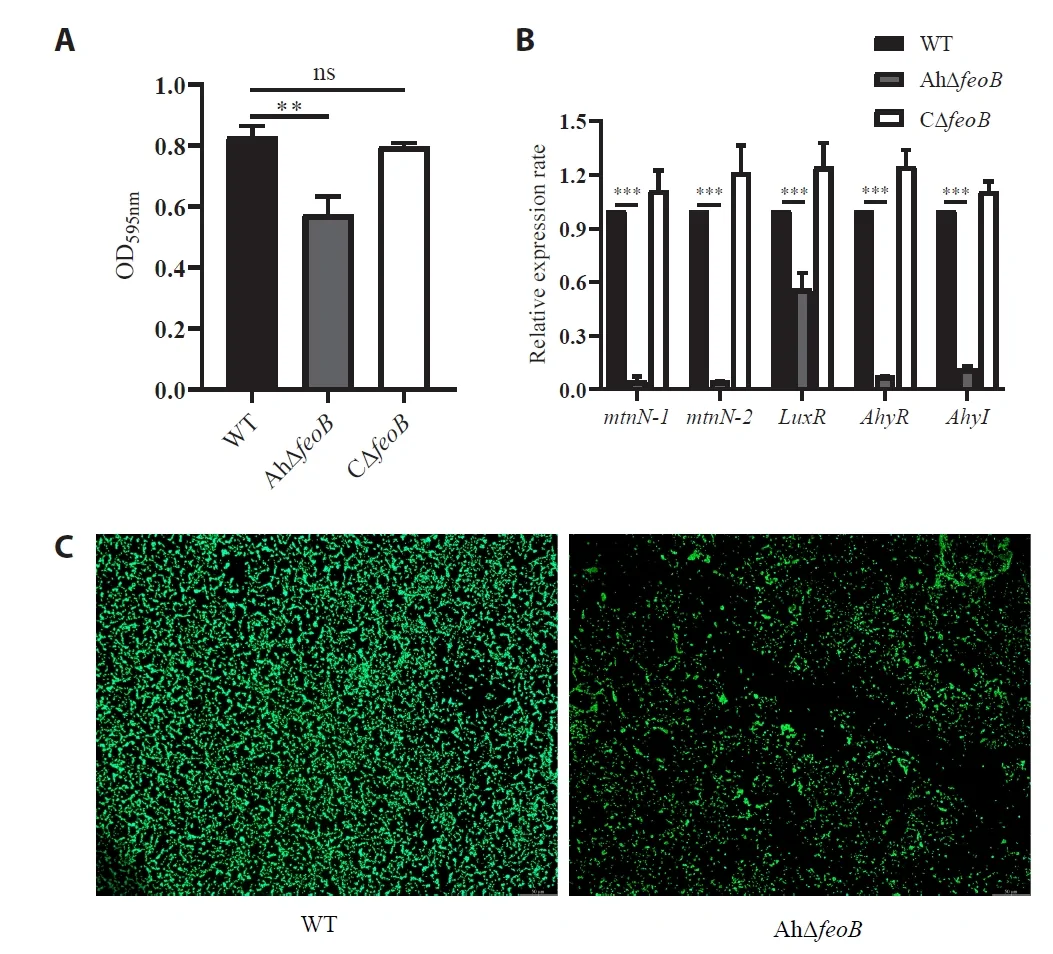
Biofilm formation of WT and AhΔ*feoB* and CΔ*feoB*. (A) Quantitative biofilm formation capacity. (B) Biofilm-associated genes mRNA expression level. (C) Acridine orange-stained fluorescence microscopy images. Data are presented as Mean ± SD. *P < 0.05, **P < 0.01, ***P < 0.001; ns, not significant; SD, standard deviation.

**Fig. 6.**
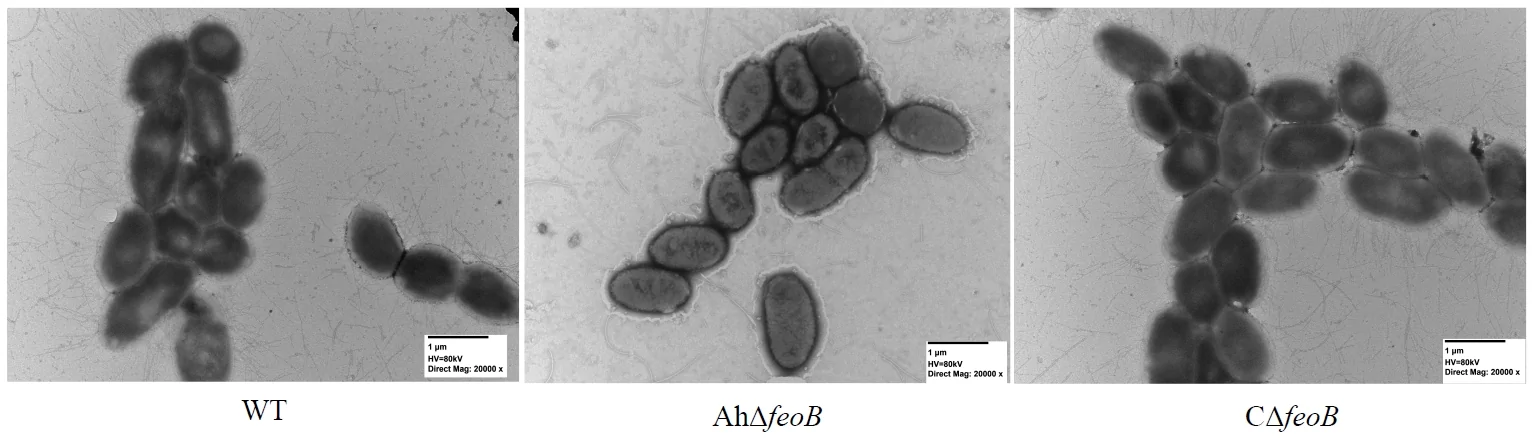
Transmission electron microscopy (TEM) observation of WT and AhΔ*feoB* and CΔ*feoB*.

**Fig. 7.**
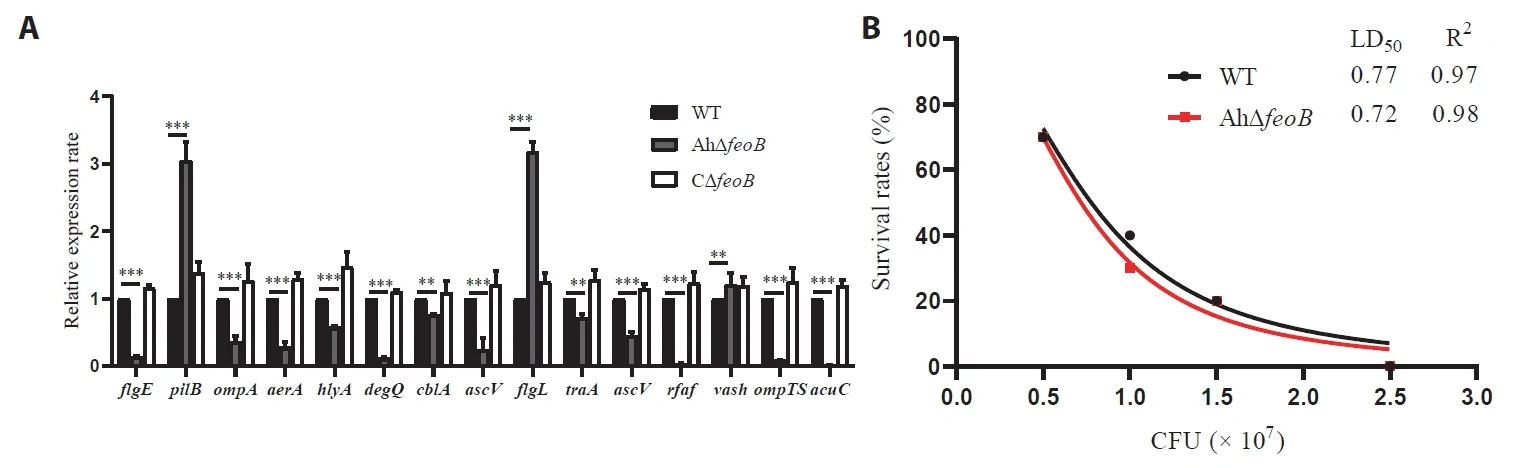
Expression levels of virulence-related genes and dose-dependent survival curves of BALB/c mice. (A) Transcript levels of virulence genes between WT and AhΔ*feoB* and CΔ*feoB*. (B) Dose-dependent survival curves of BALB/c mice infected with different doses of WT and AhΔ*feoB* cells. 50% Lethal dose (LD_50_) and R^2^ values are indicated. *P < 0.05; **P < 0.01; ***P < 0.001. ns, not significant.

**Fig. 8.**
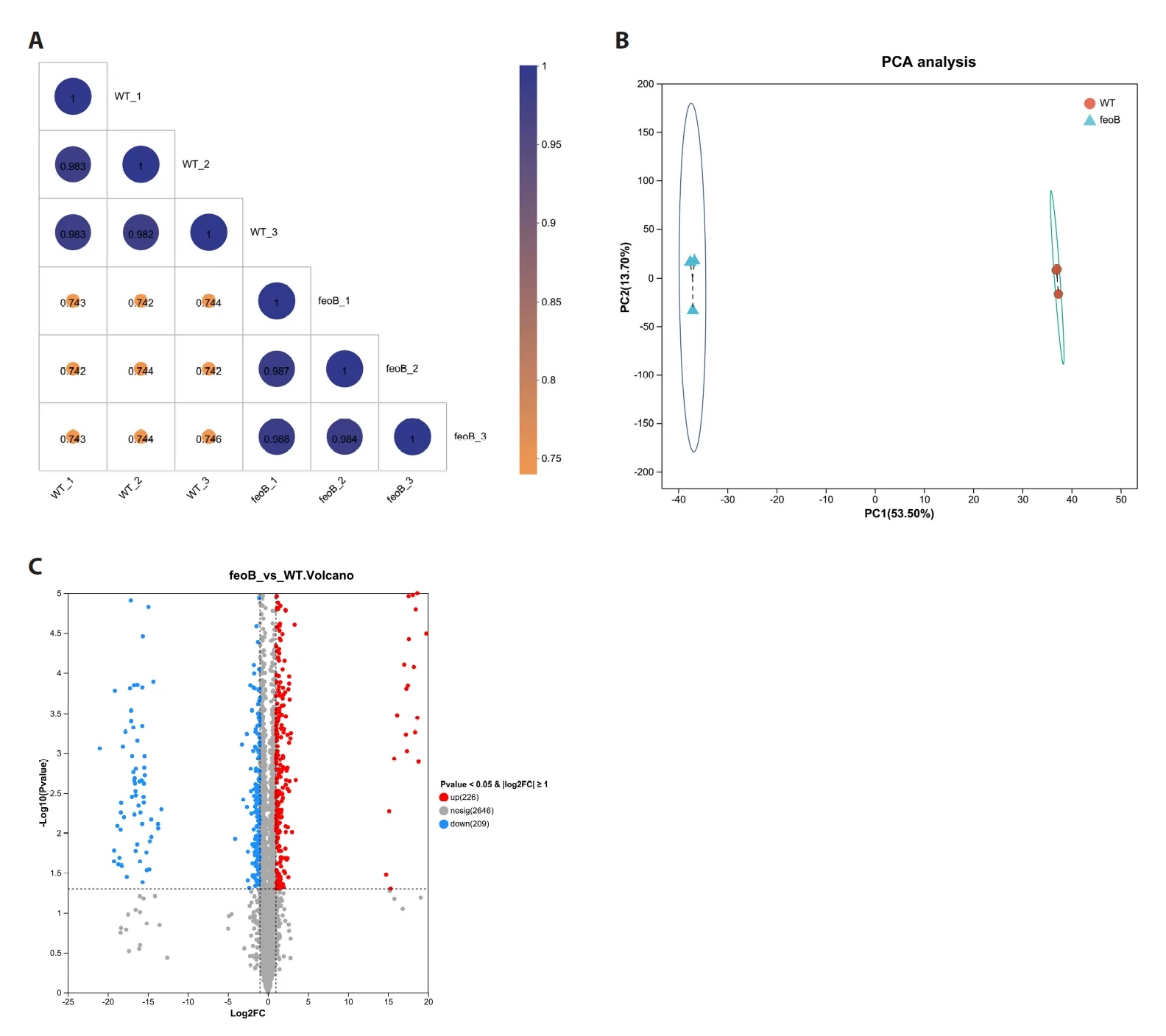
DIA-based quantitative proteomic analysis. (A) Proteins correlation analysis of three biological replicates. (B) PCA showing proteomic variations between experimental groups. (C) Volcano plot of proteins exhibiting significant differential expression (DEPs, fold change > 1, *P* < 0.05). Blue, downregulated proteins; red, upregulated proteins; gray, non-significant proteins.

**Fig. 9.**
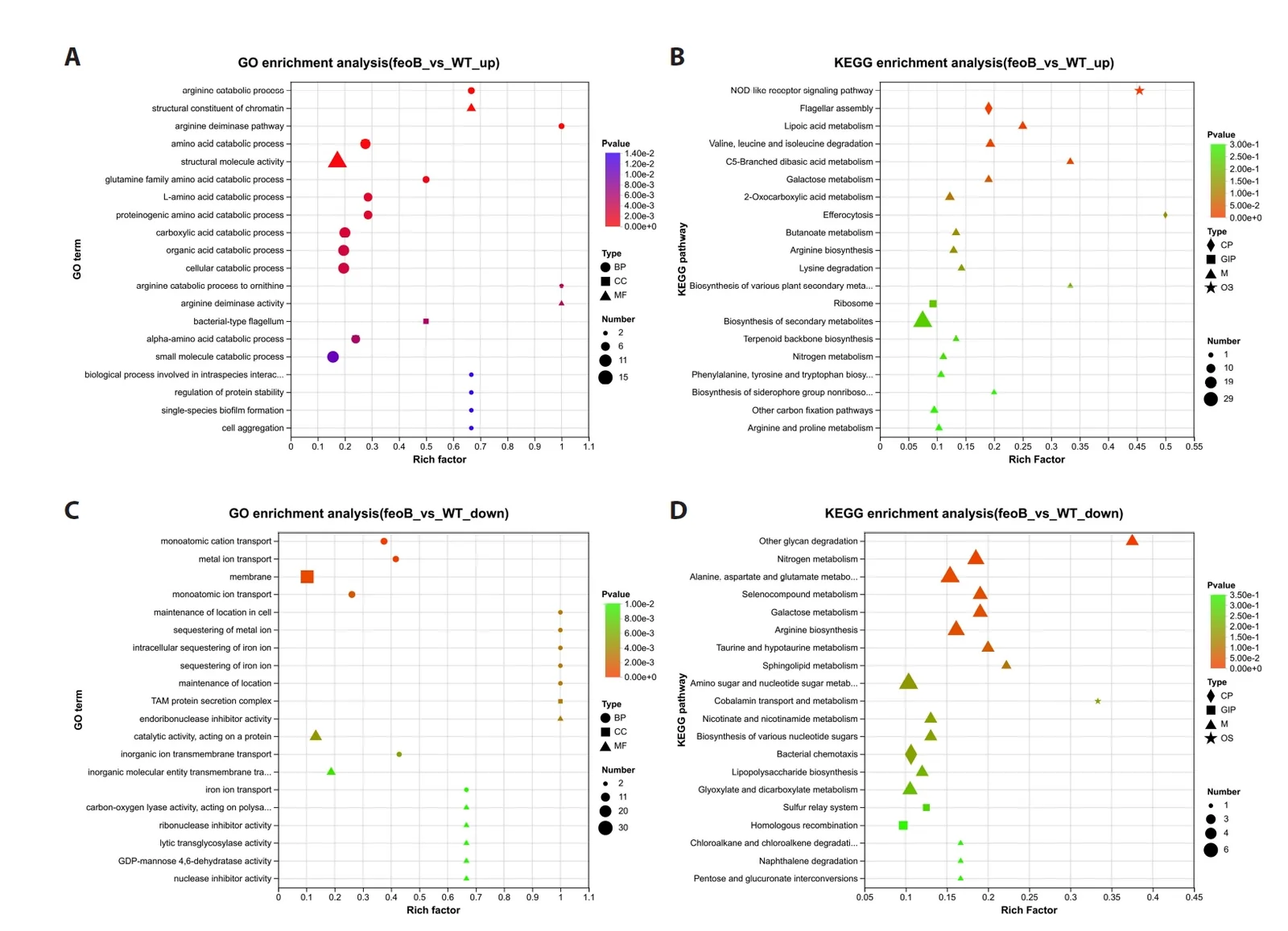
GO and KEGG enrichment bubble plots of DEPs. (A) GO enrichment of upregulated genes; (B) KEGG enrichment of upregulated genes; (C) GO enrichment of downregulated genes; (D) KEGG enrichment of downregulated genes. Bubble color corresponds to *P* value, bubble size reflects the count of enriched proteins, and the horizontal axis shows the Rich factor.

**Fig. 10.**
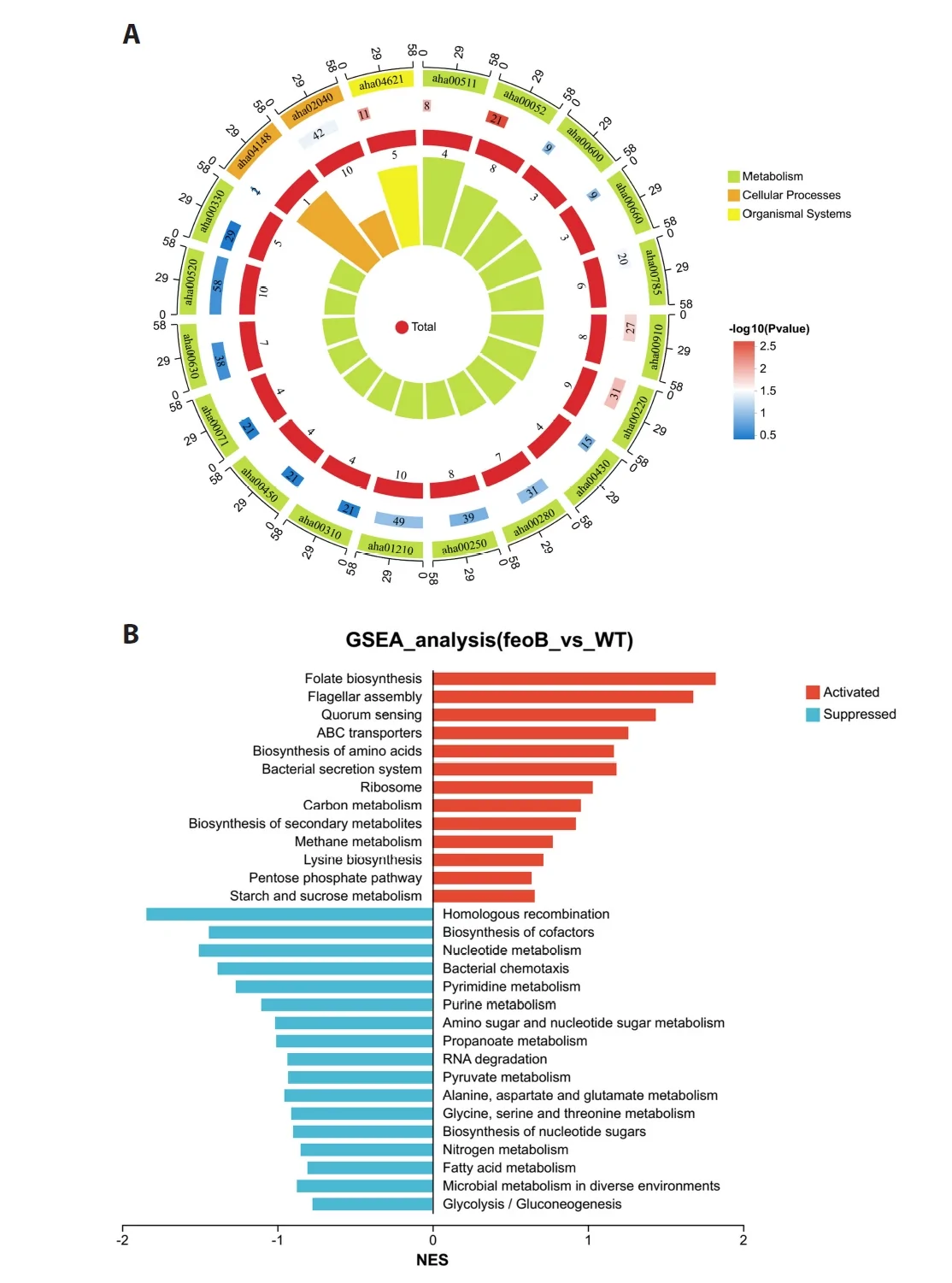
Circular enrichment analysis and GSEA analysis. (A) Circular enrichment analysis of DEPs. The four concentric layers represent the functional classification and DEP number (outermost), background protein quantity and enrichment significance (color gradient indicates P-value, redder color means more significant), the ratio of upregulated (red) and downregulated (blue) proteins, and the Rich Factor reflecting pathway enrichment degree (innermost). (B) GSEA analysis of DEPs.

**Table 1.** Strains and plasmids

Strains	Description	Source
*Aeromonas hydrophila* ATCC 7966	Wild-type strain	Laboratory preservation
*Escherichia coli* DH5α	F^−^ Ф80*lac*ZΔM15 Δ(*lac*ZYA-*arg*F) U169 *rec*A1* end*A1*hsd*R17 *pho*A *sup*E44 λ^-^thi^-^1 *gyr*A96 *rel*A1	Laboratory preservation
*Escherichia coli* S17-λpir	RP4-2(Km::Tn7,Tc::Mu-1), *pro*-82, LAMpir, *rec*A1, *end*A1, *thi*E1, *hsd*R17, *cre*C510	Laboratory preservation
AhΔ*feoB*	*A. hydrophila* ATCC 7966, in-frame deletion of *feoB*	This study
CΔ*feoB*	AhΔ*feoB* complemented with intact *feoB *gene	This study
**Plasmids**	**Description**	**Source**
pRE112	pGP704 suicide plasmid, *pir* dependent, *ori*T, *ori*V, *sac*B, Cm^r^	Laboratory preservation
pBBR1MCS1	Broad-host-range plasmid, Cm^r^	Laboratory preservation
pAR1	pRE112 harboring 446 bp upstream gene and 568 bp downstream gene of *feoB* (in-frame deletion), Cm^r^	This study
pBR-*feoB*	pBBR1-MCS1 derivative containing full fragment of *feoB* with its putative promoter and ORF, Cm^r^	This study

**Table 2.** Result of antibiotic susceptibility testing by K-B disk diffusion method

Antibiotic agent	Antibiotic class	Drug content (μg/tablet)	R/mm	I/mm	S/mm	Inhibition zone diameter (mm)/sensitivity	
						WT	AhΔ*feoB*
β-Lactams	Cefazolin	30	≤ 14	15–17	≥ 18	0/R	0/R
	Penicillin	10	≤ 14	15–19	≥ 20	0/R	0/R
	Ampicillin	10	≤ 17	18–20	≥ 21	0/R	0/R
Coumarins	Novobiocin	30	≤ 12	13–16	≥ 17	8/R	10/R
Sulfonamides	Trimethoprim-Sulfamethoxazole (SMX-TMP)	25	≤ 10	11–15	≥ 16	17/S	19/S
Macrolides	Acetylspiramycin	30	≤ 13	14–17	≥ 18	0/R	0/R
Polypeptides	Vancomycin	30	≤ 14	15–16	≥ 17	0/R	0/R
	Teicoplanin	30	≤ 10	11–13	≥ 14	0/R	0/R
	Polymyxin B	300 units	≤ 8	9–12	≥ 12	13/S	12/S
Aminoglycosides	**Streptomycin**	10	≤ 11	12–14	≥ 15	9/R	12/I
	**Gentamicin**	10	≤ 12	13–14	≥ 15	11/R	15/S
	Amikacin	30	≤ 14	15–16	≥ 17	16/I	15/I
	**Neomycin**	30	≤ 12	13–16	≥ 17	11/R	13/I
Quinolones	Ofloxacin	5	≤ 12	13–15	≥ 16	22/S	22/S
	Norfloxacin	30	≤ 12	13–16	≥ 17	20/S	23/S
	Levofloxacin	5	≤ 13	14–16	≥ 17	19/S	25/S
	**Enrofloxacin**	5	≤ 16	17–22	≥ 23	22/I	24/S
Lincosamides	Clindamycin	2	≤ 14	15–18	≥ 19	0/R	15/R
	Erythromycin	15	≤ 13	14–22	≥ 23	10/R	9/R
Tetracyclines	Tetracycline	30	≤ 11	12–22	≥ 23	17/I	20/I
	Florfenicol	30	≤ 12	13–17	≥ 18	23/S	26/S
	Minocycline	30	≤ 12	13–22	≥ 23	15/I	17/I
	Doxycycline	30	≤ 10	11–15	≥ 16	21/S	19/S
Lincosamides	Lincomycin	2	≤ 14	15–20	≥ 21	0/R	0/R
Carbapenems	Meropenem	10	≤ 19	20–22	≥ 23	21/I	19/R

R, resistant; I, intermediate; S, susceptible.

## Supplementary Information

Table S1. Sequences of primers used in this study

Table S2. Primers designed for qRT-PCR

Fig. S1. Swimming (0.25%) and swarming (0.35%) abilities of WT, AhΔ*feoB* and CΔ*feoB*. Distances were measured from the inoculation point.

Fig. S2. Oxidative stress sensitivity of WT, AhΔ*feoB* and CΔ*feoB*. Distances were measured from the inoculation point.

Fig. S3. Comparison of bacterial biofilm formation ability of WT, AhΔ*feoB* and CΔ*feoB*.

Fig. S4. Cluster analysis of differentially expressed proteins.

Fig. S5. Cluster analysis of DEPs. C1-C10: The left side shows a line plot of the expression trend of DEGs in each cluster of the all samples. The left side of the rectangle shows the serial number of clusters, and the number of genes within each cluster (cluster size) is shown below the line plot. In the middle is a heat map of the clusters of DEGs in the WT and *feoB* samples. The right side shows the pathways that were significantly enriched for DEGs in each cluster after GO enrichment analysis.

Fig. S6. Linear relationship between iron concentration and OD_593_.

Fig. S7. The protein assay standard curve for BCA.
