# Supplementary material for: Contribution of ferrous iron transporter FeoB to iron utilization, membrane stability and biofilm formation in Aeromonas hydrophila
Source: J Microbiol. 2026 Oct 31;64(10):e2605004. doi: 10.71150/jm.2605004 (PMC13720380; doi:10.71150/jm.2605004)
Supplement: Supplementary file 1 [file jm-2605004-Supplementary-Fig-S1.pdf]

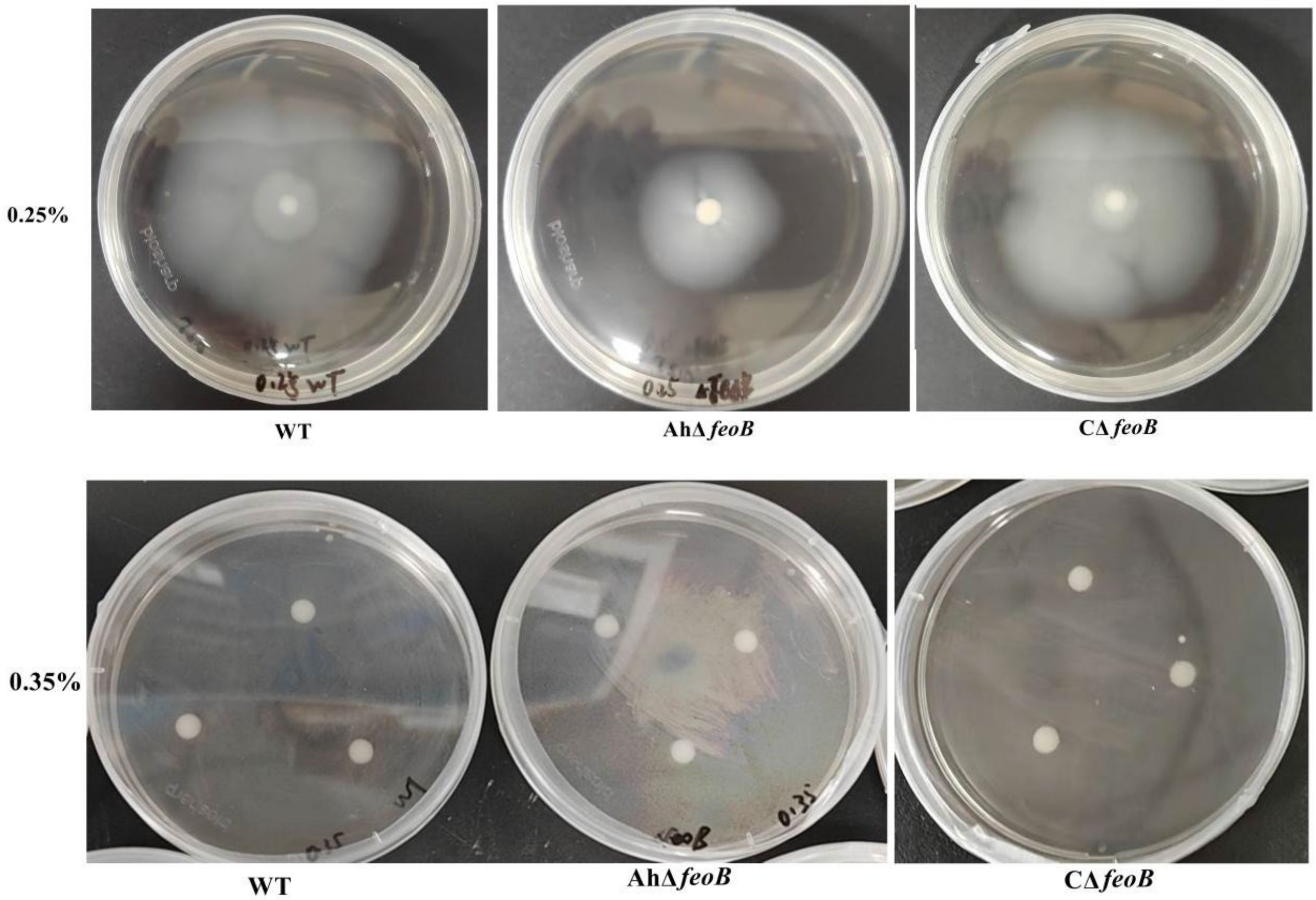

Fig. S1. Swimming (0.25%) and swarming (0.35%) abilities of WT, AhΔ*feoB* and CΔ*feoB*. Distances were measured from the inoculation point.
