# Supplementary material for: Contribution of ferrous iron transporter FeoB to iron utilization, membrane stability and biofilm formation in Aeromonas hydrophila
Source: J Microbiol. 2026 Oct 31;64(10):e2605004. doi: 10.71150/jm.2605004 (PMC13720380; doi:10.71150/jm.2605004)
Supplement: Supplementary file 2 [file jm-2605004-Supplementary-Fig-S2.pdf]

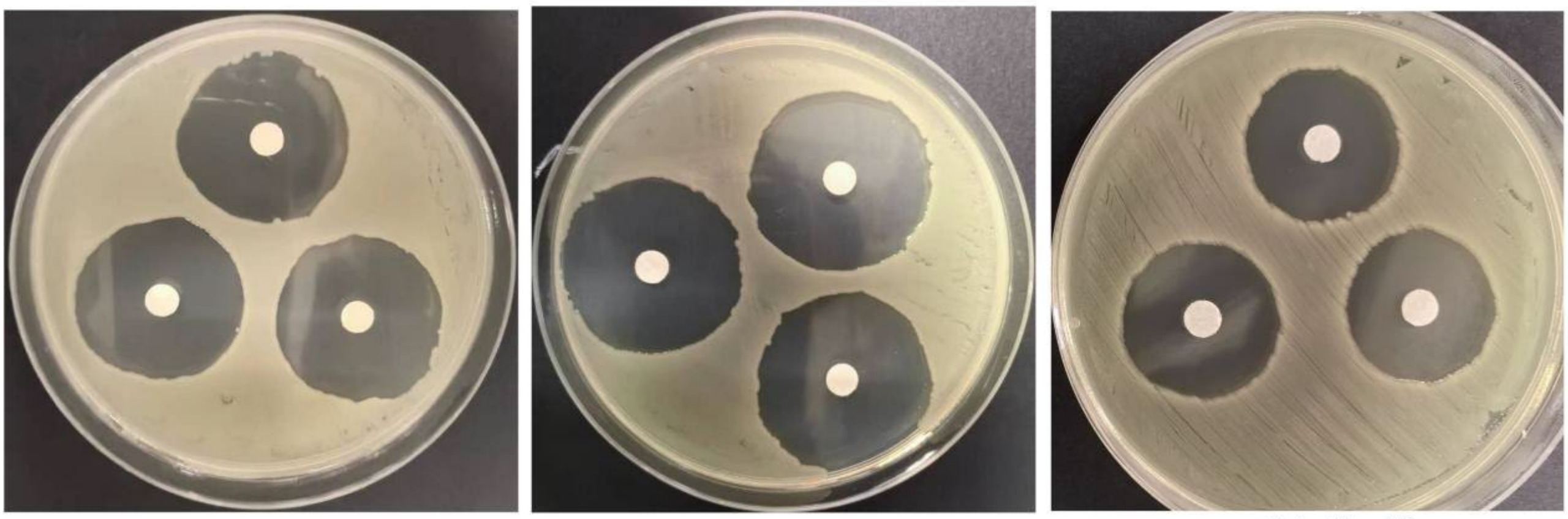

WT **Ah∆*feoB*** **C∆*feoB***

Fig. S2. Oxidative stress sensitivity of WT, Ah∆*feoB* and C∆*feoB*. Distances were measured from the inoculation point.
