# Supplementary material for: Contribution of ferrous iron transporter FeoB to iron utilization, membrane stability and biofilm formation in Aeromonas hydrophila
Source: J Microbiol. 2026 Oct 31;64(10):e2605004. doi: 10.71150/jm.2605004 (PMC13720380; doi:10.71150/jm.2605004)
Supplement: Supplementary file 4 [file jm-2605004-Supplementary-Fig-S4.pdf]

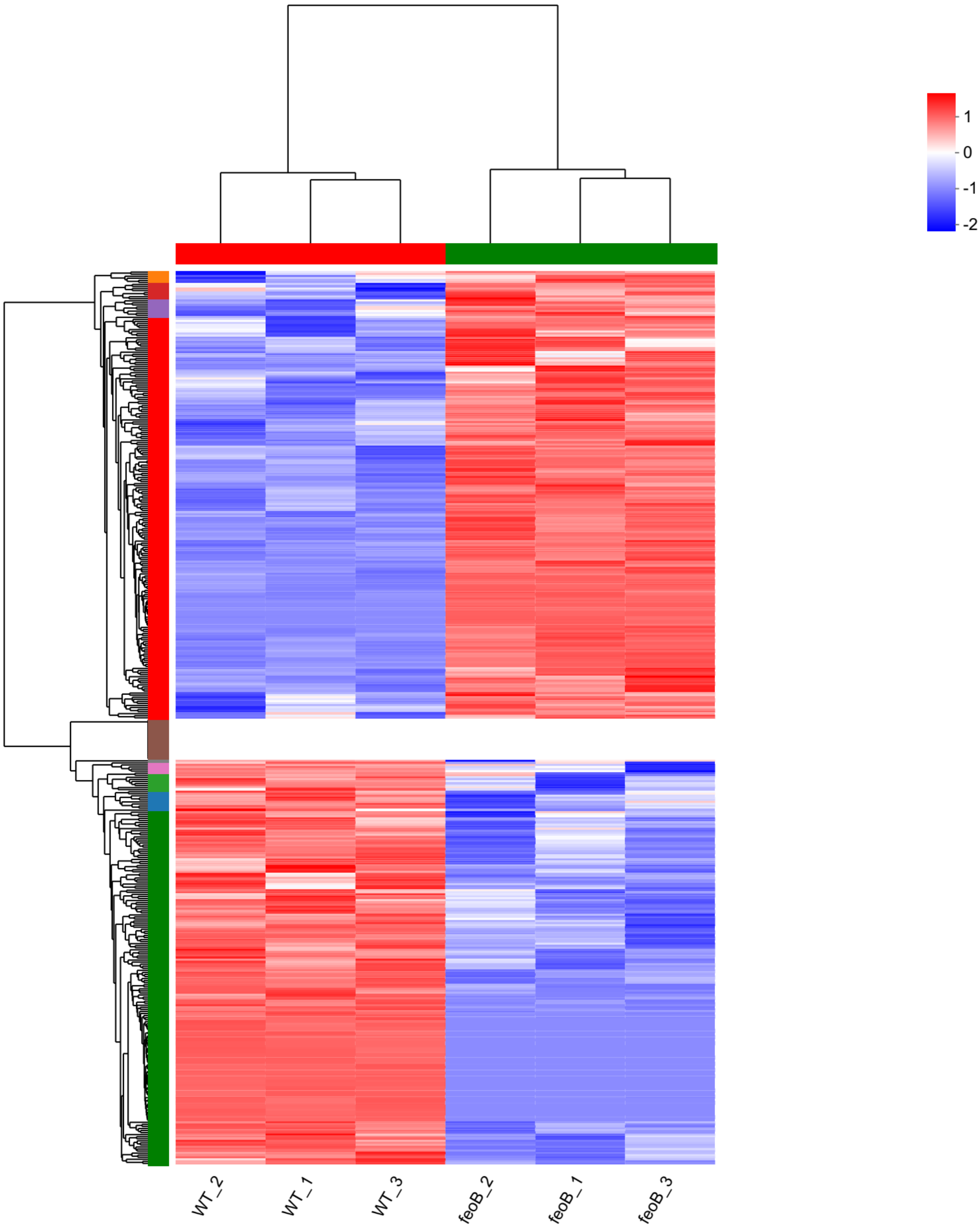


**Fig. S4.** Cluster analysis of differentially expressed proteins.
