# Supplementary material for: Contribution of ferrous iron transporter FeoB to iron utilization, membrane stability and biofilm formation in Aeromonas hydrophila
Source: J Microbiol. 2026 Oct 31;64(10):e2605004. doi: 10.71150/jm.2605004 (PMC13720380; doi:10.71150/jm.2605004)
Supplement: Supplementary file 5 [file jm-2605004-Supplementary-Fig-S5.pdf]

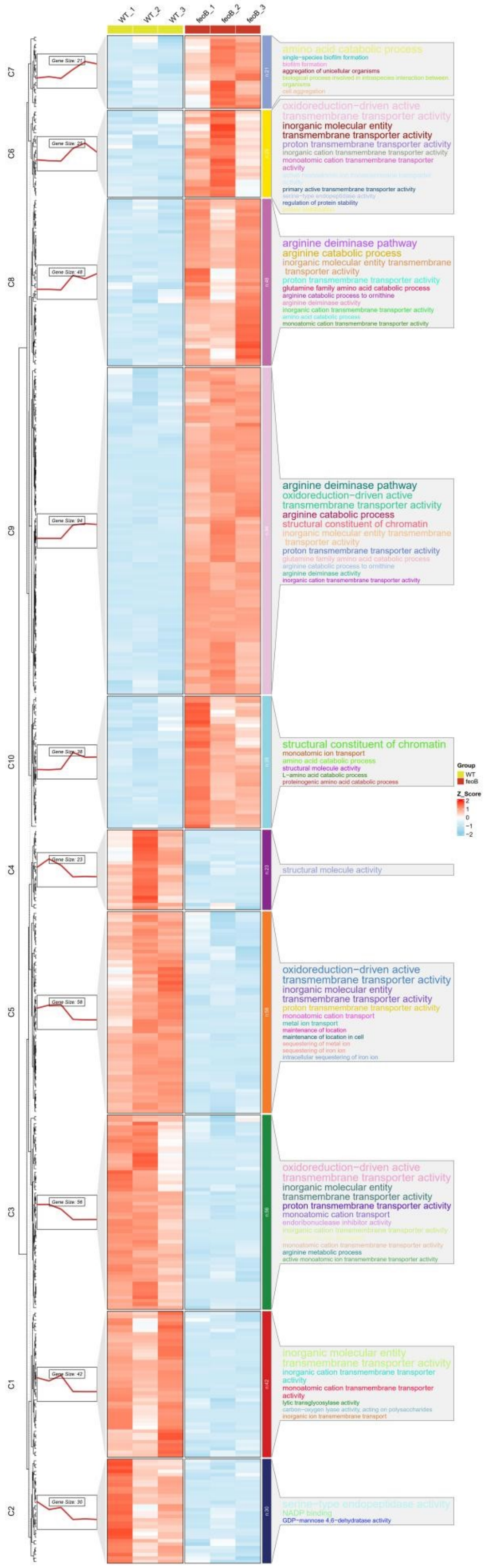

Fig. S5. Cluster analysis of DEPs. C1-C10: The left side shows a line plot of the expression trend of DEGs in each cluster of the all samples. The left side of the rectangle shows the serial number of clusters, and the number of genes within each cluster (cluster size) is shown below the line plot. In the middle is a heat map of the clusters of DEGs in the WT and *feoB* samples. The right side shows the pathways that were significantly enriched for DEGs in each cluster after GO enrichment analysis.
