# Supplementary material for: Contribution of ferrous iron transporter FeoB to iron utilization, membrane stability and biofilm formation in Aeromonas hydrophila
Source: J Microbiol. 2026 Oct 31;64(10):e2605004. doi: 10.71150/jm.2605004 (PMC13720380; doi:10.71150/jm.2605004)
Supplement: Supplementary file 8 [file jm-2605004-Supplementary-Table-S1.pdf]

Table S1. Sequences of primers used in this study

**Target genes**	**Primer**	**Sequence (5'→3')**
Upstream region of *feoB*	P1-up (*Xba*I)	CTCTAGATGGTCACCGGCGGTGGA
	P1-down (*Sac*I)	CGAGCTCTCCTGCGTGAGCTGGGG
Downstream region of *feoB*	P2-up (*Xba*I)	CGAGCTCACCGGTTCAACCTCGATAG
	P2-down (*Nde*I)	CCATATGCGCCGTGGATGGTGATG
External region of *feoB* for identification	P3-up	CCTTACGCGAACACAGC
	P3-down	CTTCAGGAGTCAGTCATGGT
feoB for qRT-PCR	*feoB*-qF	TGCTCAACACCGAGCTTTCT
	feoB-qR	GAGATGCCGGACTACGAGTG
feoB with ORF	*feoB*-F (*Xba*I)	CTCTAGAATGAACTACTCCCTGGTAACCGTC
	feoB-R (*Hind*III)	CAAGCTTTCACAGGGTCACCACCTTGC
