# Supplementary material for: Contribution of ferrous iron transporter FeoB to iron utilization, membrane stability and biofilm formation in Aeromonas hydrophila
Source: J Microbiol. 2026 Oct 31;64(10):e2605004. doi: 10.71150/jm.2605004 (PMC13720380; doi:10.71150/jm.2605004)
Supplement: Supplementary file 9 [file jm-2605004-Supplementary-Table-S2.pdf]

Table S2. Primers designed for qRT-PCR

Gene	Primer	Sequence (5'→3')
tonB1	tonB1-F	ACGGTTTTTAAGCGGTTTG
	tonB1-R	CGCCTTCTGGTGGCTCTAT
tonB2	tonB2-F	CCTCGACCACTTCCACATCC
	tonB2-R	GCGAGCGGCAACTTCCAT
tonB3	tonB3-F	CGATGGACATGCCCAAACTC
	tonB3-R	GGTGAACTCCACCAGCACCTT
acuC	Ac-F	AGGACGATGCCTACCTCACC
	Ac-R	GCGTTTGTTCGCCTCTTCA
cblA	Ad-F	CCGAGGCGTTCTATGTGCA
	Ad-R	TTGGTCAGGTAGCCGGTGAT
aerA	Ae-F	GGTCTGTGGCGACAAGTATCG
	Ae-R	AGAGCAGACAGAGTCGGTATTTCTC
ascV	As-F	GGGTATTCACCTGCGTTTCA
	As-R	GATGTTCATTAGCGACCCACA
hlyA	Hl-F	CCGCCCAGTCCTTCATCTAT
	Hl-R	AGGGTCCGTAGGCTCACATT
ompA	Om-F	CTCACGATCTGGGTGACTTTG
	Om-R	CGCCGTTGATGGACTTGA
ompTS	Pr-F	AATGGCTCCTTCCCTGATCG
	Pr-R	TGGCACCCTGGTTCTCGTAA
vash	Va-F	AAACTGGCACGGGGAAAGAG
	Va-R	GCTTGTAAGGTGAGCGGCATAT
traA	Tr-F	GTGATGGTCGTCGCCTTTCT
	Tr-R	GATAACCTTCTCCGCATTTTCC
pilB	Pi-F	CTAATGCGAATGCAGCACGTA
	Pi-R	CGCTTCAACAGTTCCAACCA
rfaf	Rf-F	TACCTGGCACTGGCCTATCC
	Rf-R	CTCGTCGAGGTGCTTTTGTG
degQ	De-F	CACCGAGCTTACCTCCGAAAT
	De-R	CCGCCTTCTTCAGCGTGAC
flgE	Fe-F	CCCGCTCAGACATTGGAGAT
	Fe-R	GTCGCATTGCTGTAGGTCGC
flgL	Fl-F	GCCCCAGAACAACAACATCC
	Fl-R	GCCGCATCCTCTTTTGACA
mtnN-1	Mt1-F	AGGTCTGATGCAGCCAGTT
	Mt1-R	TGGCACAACTTCGAGGTCA

mtnN-2	Mt2-R	TGATCGCCGGTGCAGATGA
	Mt2-R	TGATCGCCAGCGAGATG
AhyI	Ah-F	AGATGCTGGGCAGCATGTA
	Ah-R	TGGAGCAGGACAGTTTCGATA
AhyR	Ay-F	ACGGCATCATACTGTCGTTCATCAC
	Ay-R	CCTCGAAGATGTAGTTGGACATCCA
LuxR	Lu-F	ACCGGCTTCTACATGAG
	Lu-R	ATGGGCCTCTTCCAGTGA
16S rDNA	16s-F	GGGGAGTACGGTCGCAAGAT
	16s-R	CGCTGGCAAACAAGGATAAGG
